# Severe COVID‐19 Unveils Atypical Familial Hemophagocytic Lymphohistiocytosis due to a Novel Homozygous *PRF1* Variant

**DOI:** 10.1155/crii/3879317

**Published:** 2026-02-10

**Authors:** Nasimeh Vatandoost, Mohsen Jari, Mansour Salehi

**Affiliations:** ^1^ Department of Genetics and Molecular Biology, Medical School, Isfahan University of Medical Sciences, Isfahan, Iran, mui.ac.ir; ^2^ Pediatric Inherited Diseases Research Center, Research Institute for Primordial Prevention of Non-Communicable Disease, Isfahan University of Medical Sciences, Isfahan, Iran, mui.ac.ir; ^3^ Cellular, Molecular, and Genetics Research Center, Isfahan University of Medical Sciences, Isfahan, Iran, mui.ac.ir; ^4^ Department of Pediatric Rheumatology, Imam Hossein Children’s Hospital, Isfahan University of Medical Sciences, Isfahan, Iran, mui.ac.ir

## Abstract

We report a novel homozygous *PRF1* variant, *PRF1* (NM_001083116.3):c.343G > A (p.Glu115Lys), identified by whole‐exome sequencing (WES) in an 11‐year‐old girl with atypical familial hemophagocytic lymphohistiocytosis (FHL). The variant, inherited from asymptomatic heterozygous parents, was absent or extremely rare in population databases and was predicted to be deleterious by multiple in silico tools. Born to consanguineous parents, the patient presented with recurrent fever, pancytopenia, and multiorgan failure following SARS‐CoV‐2 infection, further complicated by Epstein–Barr virus (EBV) and cytomegalovirus (CMV) coinfections. Despite intensive immunosuppressive therapy, she developed seizures, an intracranial hemorrhage, and died at age 11. A striking family history of unexplained febrile deaths in infancy and childhood strongly supported autosomal recessive inheritance. It emphasizes the role of viral triggers, especially COVID‐19, in revealing genetic predispositions and underscores the importance of genetic screening in atypical cases.

## 1. Introduction

Hemophagocytic lymphohistiocytosis (HLH) is a life‐threatening hyperinflammatory syndrome caused by uncontrolled immune activation [1]. Primary familial HLH (FHL) typically presents in infancy due to biallelic pathogenic variants in genes encoding cytotoxic proteins, including *PRF1*, *UNC13D*, *STX11*, and *STXBP2* [2–5]. However, late‐onset and adult presentations are increasingly recognized [6–8]. The *PRF1* gene encodes perforin, essential for pore formation and target‐cell killing by NK cells and cytotoxic T lymphocytes. Loss‐of‐function variants in *PRF1* (FHL2) account for 20%–50% of FHL cases [9]. Recently, SARS‐CoV‐2, the virus responsible for COVID‐19, has emerged as a potent trigger for HLH. It exacerbates underlying genetic vulnerabilities, thereby complicating diagnosis and treatment. The atypical presentations pose significant diagnostic and therapeutic challenges because of shared symptoms with other autoimmune and hyperinflammatory disorders [10–12].

We report an 11‐year‐old patient whose condition worsened after COVID‐19 and showed signs of HLH. Whole‐exome sequencing (WES) identified a novel homozygous *PRF1* mutation, which was subsequently confirmed by Sanger sequencing and cosegregation analysis. This study highlights the importance of genetic testing in atypical HLH cases and emphasizes the role of viral triggers, like SARS‐CoV‐2, in revealing genetic predispositions to hyperinflammatory conditions. Our findings expand the known *PRF1* mutations associated with HLH and underscore the need for personalized diagnosis and treatment in such complex cases.

## 2. Materials and Methods

### 2.1. Case Presentation and DNA Extraction

The patient’s medical history, clinical data, and family pedigree were documented following genetic counseling. Peripheral blood was collected with informed consent from the legal guardian, and the study was approved by the Ethics Committee of Isfahan University of Medical Sciences (IR.MUI.MED.REC.1401.006). Genomic DNA was extracted using the FAVORGEN Blood Genomic DNA Extraction Mini‐Kit (Tehran, Iran).

### 2.2. WES and Variant Prioritization

WES was performed on the proband’s DNA using the Illumina NovaSeq x 10B platform (2 × 150 bp, 300‐cycle configuration) with the xGen Exome Research Panel v2 (Integrated DNA Technologies). Sequencing achieved a mean depth of 100x, with over 90% of target bases at 20x coverage and over 95% at a Q30 quality score. Reads were aligned to GRCh38 using BWA‐MEM, and variants were called with GATK HaplotypeCaller. Variants were prioritized and interpreted in accordance with the ACMG/AMP guidelines [13].

### 2.3. In Silico and Structural Analysis

Variant pathogenicity was evaluated using SIFT, PolyPhen‐2, MutationTaster, CADD, FATHMM‐MKL, and BayesDel, with allele frequencies checked in gnomAD v2.1.1, Iranome, and GTEx databases. Conservation analysis of the p.(Glu115Lys) variant within the MACPF domain was performed using Clustal Omega v1.2.4 (https://www.ebi.ac.uk/tools/msa/clustalo/) with default settings (including multiple sequence alignment iterations and four iterations), and the results were visualized using Jalview v2.11.1.5. Protein stability was assessed with INPS‐3D, mCSM, DUET, DDGun, SDM, SAAFEC‐SEQ, and I‐Mutant2.0. Homology models were built using SWISS‐MODEL (https://swissmodel.expasy.org) with template PDB 3NSJ and visualized in PyMOL v3.1.6.1 (https://pymol.org).

### 2.4. Sanger Sequencing and Cosegregation Analysis

Sanger sequencing confirmed the candidate mutation, and cosegregation analysis was performed on the family. Primers (*PRF1*‐F/R) were designed to amplify a 540‐bp region containing the variant and were validated using online tools, including Primer‐BLAST and SNP Check (Gene Tools, SNP Check V3). The primer sequences used are listed in Table 1.

**Table 1:** Primers used for Sanger sequencing.

Primer name	Sequence (5′–3′)	Product size (bp)
*PRF1*‐F	ATTCCAGAGCCCAAGTGC	—
*PRF1*‐R	GCTGAAGCTGTACTGGTCC	540

## 3. Result

### 3.1. Subject

An 11‐year‐old girl, born to consanguineous parents, had an unremarkable prenatal and postnatal history with normal development until age 6. She experienced minor, unexplained fevers that resolved on their own until she developed abdominal pain and fever at age 6, initially attributed to stress. After contracting COVID‐19 at age 6, she presented with high‐grade fever, pancytopenia, and splenomegaly confirmed by ultrasound. Laboratory results showed elevated liver enzymes (ALT:171 U/L and AST:395 U/L), borderline thrombocytopenia (30 × 10^9^/L), increased triglycerides (2.99 mmol/L), and high ferritin levels (638.8 µg/L) (Table 2). She was hospitalized repeatedly every 1–2 months with symptoms of fever, joint pain, and cytopenia, fulfilling part of the HLH diagnostic criteria [14]. She was initially started on intravenous immunoglobulin (IVIG) for its anti‐inflammatory effects due to an uncertain diagnosis and concerns about the potential for worsening infections. Additionally, high‐dose intravenous glucocorticoids were initiated. A few months later, the patient began to report progressive muscular weakness in her lower limbs, leading to frequent falls. During her hospitalization, she developed seizures, and a repeat head CT scan showed a new intracranial hemorrhage with herniation. At this point, she was treated with high doses of intravenous glucocorticoids, cyclosporin A, and dexamethasone. Throughout her hospitalization, she also required supportive therapy, including leukocyte‐depleted red blood cells, platelet units, and frozen plasma. Serological testing confirmed Epstein–Barr virus (EBV; IgM‐positive, 3328 DNA copies/mL) and cytomegalovirus (CMV;116.9 copies/mL) infections, with retinal inflammation causing severe vision loss. Despite 4 years of treatment, the patient died at age 11, just before planned intrathecal chemotherapy. The family medical history was notable for the deaths of five cousins who suffered from undiagnosed illnesses characterized by prolonged fever. All of these cousins died during their first or second year of life.

**Table 2:** Laboratory findings across the patient’s clinical course.

Parameter	Initial lab results	Monitoring during periods of disease inactivity	Fever + pancytopenia (admission)	Discharge	Significant fever episode	Reference range
Hemoglobin (× 10^9^/L)	1.58 (L)	7.95	0.81 (L)	2.56 (L)	1.80 (L)	108–133
Platelet (× 10^9^/L)	33 (L)	136 (L)	30 (L)	46 (L)	24 (L)	194–345
WBC (× 10^9^/L)	—	—	—	—	—	4.5–13.5
Neutrophile (× 10^9^/L)	0.80 (L)	3.98	0.26 (L)	1.80 (L)	1.09 (L)	1.82–7.47
Lymphocytes (× 10^9^/L)	0.90 (L)	2.54	0.35 (L)	0.49 (L)	0.37 (L)	1.16–3.33
Monocytes (× 10^9^/L)	0.46	0.80 (H)	0.11 (L)	0.24 (L)	0.08 (L)	0.19–0.72
Eosinophil (× 10^9^/L)	0.05	0.24	0.03 (L)	0.01 (L)	0.00 (L)	0.02–0.32
Basophilia (× 10^9^/L)	0.00 (L)	0.00 (L)	0.00 (L)	0.01	0.00 (L)	0.01–0.05
Alanine transaminase(U/L)	28	‐	171 (H)	‐	127 (H)	< 31
Aspartate aminotransferase (U/L)	17	‐	395 (H)	‐	331 (H)	< 31
Fibrinogen(g/L)	—	2.6	2.0	—	1.5 (L)	1.9–4.3
Ferritin (µg/L)	351.4 (H)	239.6 (H)	638.8 (H)	687.6 (H)	4873.6 (H)	13.7– 78.8
Triglycerides (mmol/L)	‐	2.38 (H)	2.99 (H)	2.06 (H)	5.49 (H	< 1.02
CRP (mg/L)	‐	‐	‐	108.8	200	< 10
E.S.R 1 h	15 mm/h	—	—	—	—	—
CMV qualitative PCR	‐	‐	‐	‐	Positive‐116.9 copies/mL	Negative
EBV qualitative PCR	‐	‐	‐	‐	Positive‐3328 copies/mL	Negative

*Note*: Values marked (L) or (H) indicate low or high relative to reference ranges. “Inactive period” reflects monitoring between flares. “Significant fever episode” denotes the final flare before death. Missing values are indicated by “‐”.

Additionally, the mother’s uncles also passed away due to a fever complicated by jaundice, with ages at death ranging from 1 to 20 years. The father’s brother also died because of repetitive, unexplained seizures. Unfortunately, details regarding the treatment regimens and investigations of deceased patients are unavailable (Figure 1A).

Figure 1(A) Pedigree showing autosomal recessive inheritance. The proband (II‐1, arrow) is homozygous (filled symbol) with atypical HLH and a fatal outcome; parents (I‐1 and I‐2) and sibling (II‐2) are heterozygous (half‐shaded); five cousins, two maternal uncles, and one paternal uncle (shaded with diagonal lines) had unexplained febrile deaths or seizures. (B) Sanger sequencing electropherograms confirming the *PRF1* (NM_001083116.3):c.343G > A (p.Glu115Lys) variant: homozygous in the proband, heterozygous in parents and healthy sibling, supporting cosegregation (PP1and ACMG).

**Figure:**
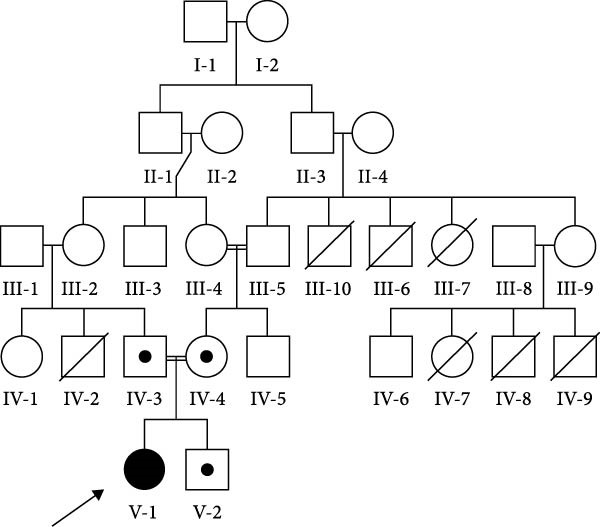
(A)

**Figure:**
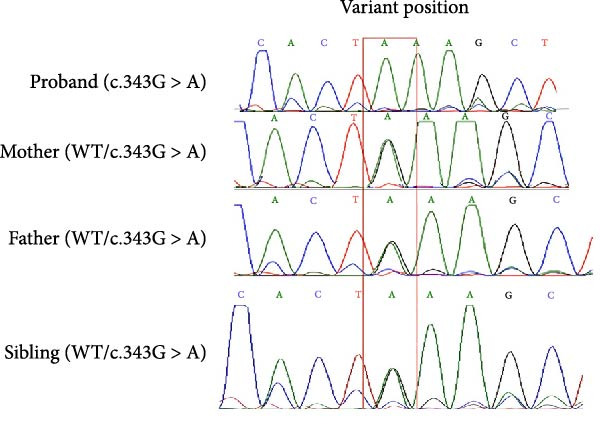
(B)

### 3.2. Genetic Findings

WES identified a homozygous variant *PRF1* (NM_001083116.3):c.343G > A (p.Glu115Lys). Sanger sequencing confirmed homozygosity in the proband and heterozygosity in both parents and the healthy sibling (Figure 1B). The variant is absent from gnomAD v4 and Iranome. All in silico tools predicted pathogenicity (Table 3). Conservation analysis identified Glu115 as conserved across species (Figure 2A). Predicted protein structural changes based on SWISS‐MODEL homology models of wild type and mutant Perforin‐1 yielded GMQE = 0.92 and QMEAN = −0.85. PyMOL v2.5 alignment yielded an RMSD of 0.054 Å across 516 of 555 residues. Visualization of NP_001076585.2:p.(Glu115Lys) in the MACPF domain’s β‐sheet, a charge reversal from glutamate to lysine, was displayed (Figure 2B). Stability predictions indicated a destabilizing effect (Table 4).

Figure 2Conservation and structural analysis of the *PRF1* p.(Glu115Lys) variant. (A) Conservation analysis of Glu115 across species, performed using clustal omega and visualized with Jalview, showing high conservation in the MACPF domain. (B) Structural visualization of the NP_001076585.2:p.(Glu115Lys) variant in the MACPF domain using PyMOL. Cartoon and surface views highlight the charge shift from glutamate (negative, green) to lysine (positive, red) in the β‐sheet, with an RMSD of 0.054 Å for aligned residues.

**Figure:**
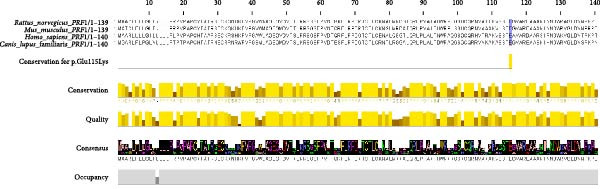
(A)

**Figure:**
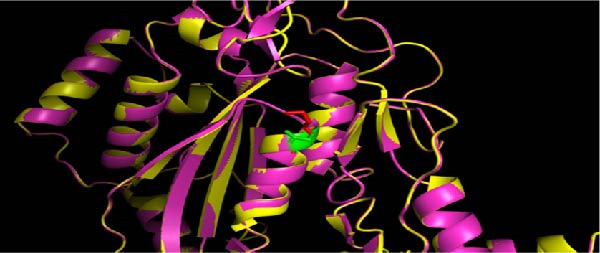
(B)

**Table 3:** In silico pathogenicity predictions for *PRF1* (NM_001083116.3):c.343G > A.

Tool/database	Score	Classification (threshold)	References
SIFT	0.02	Damaging ( ≤ 0.05)	[15]
PolyPhen‐2	0.98	Probably damaging ( ≥ 0.85)	[16]
MutationTaster	1.0	Disease‐causing (1.0)	[17]
CADD (Phred)	17.09	Deleterious ( ≥ 10)	[18]
FATHMM‐MKL	0.9223	Damaging ( ≥ 0.5)	[19]
BayesDel (addAF)	0.1077	Pathogenic ( ≥ 0.057)	[20]
gnomAD v2.1.1 1000Genomes, GME, and Iranome (allele frequency)	< 0.0001	Rare (supportive of PM2)	[21–24]

**Table 4:** Computational predictions of protein stability for *PRF1* NP_001076585.2: p.(Glu115Lys).

Stability prediction server	INPS‐3D	mCSM	DUET	DDGun	SDM	SAAFEC‐SEQ	I‐Mutant2.0
*PRF1*: (p.Glu115Lys)	Destabilizing	Destabilizing	Destabilizing	Destabilizing	Destabilizing	Destabilizing	Destabilizing

*Note*: All tools predict a destabilizing effect on the *PRF1* protein structure due to the NP_001076585.2: p.(Glu115Lys) substitution, supporting a potential loss‐of‐function.

### 3.3. ACMG Classification

The variant *PRF1* (NM_001083116.3):c.343G > A p.(Glu115Lys) is located in Exon 2 of the transcript and within the MACPF domain of the *PRF1* protein (Figure 3). It was classified as a variant of uncertain significance (VUS) in ClinVar by two laboratories (Invitae SCV002175656.3 and Ambry SCV005152109.1) based solely on its rarity and in silico predictions. We contacted both laboratories on September 18, 2025, to share phenotypic data; Invitae declined due to privacy restrictions, and Ambry did not respond. According to the full ACMG/AMP guidelines, it meets the following criteria: PM2 (allele frequency less than 0.0001 in gnomAD, 1000 Genomes, Greater Middle East [GME], and Iranome, indicating the rarity of the variant, as shown in Table 3), PM1 (located in the conserved MACPF domain, Figure 3), PP1 (cosegregation with HLH in the family, heterozygous in a healthy sibling and unaffected parents), PP3 (multiple in silico tools predict damaging effects, Table 3), and PP4 (phenotype specific for HLH).

Figure 3(A) Transcript NM_001083116.3 showing exons and c.343G > A in exon 2. (B) Protein NP_001076585.2 domains with p.(Glu115Lys) in the MACPF domain.

**Figure:**
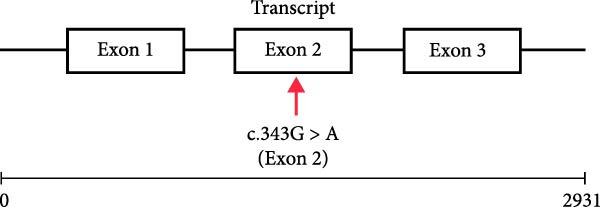
(A)

**Figure:**
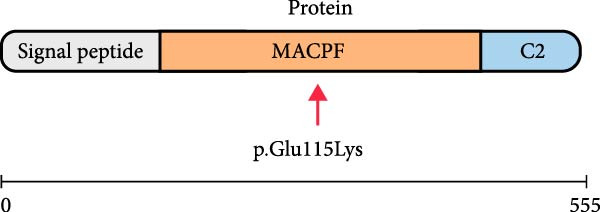
(B)

## 4. Discussion

This case describes an 11‐year‐old female with atypical HLH triggered by SARS‐CoV‐2, linked to a novel homozygous variant *PRF1* (NM_001083116.3):c.343G > A (p.Glu115Lys). The patient’s late‐onset presentation, with recurrent fevers, pancytopenia, and multiorgan dysfunction postCOVID‐19, aligns with reports of FHL manifesting beyond infancy [3, 5]. The three extensive family histories of unexplained febrile deaths and seizures (Figure 1A) support a genetic etiology, consistent with autosomal recessive inheritance confirmed by cosegregation analysis (Figure 1B).

The *PRF1* (NM_001083116.3):c.343G > A (p.Glu115Lys) variant, located in the conserved MACPF domain (Figure 2A), was predicted to be pathogenic by multiple in silico tools (Table 3), with an allele frequency of less than 0.0001 in gnomAD, supporting its rarity (PM2 and ACMG). Structural analysis with SWISS‐MODEL and PyMOL (RMSD = 0.054 Å, 516 out of 555 residues aligned) showed minimal overall structural deviation but a significant change in charge at residue 115, from glutamate (negative) to lysine (positive), within the MACPF domain’s β‐sheet (Figure 2B). This charge reversal, along with stability predictions indicating a destabilizing effect (Table 4), suggests a potential disruption of electrostatic interactions or hydrogen bonds, which are essential for perforin’s pore‐forming function. Such disruptions may impair NK cell and CTL cytotoxicity, contributing to the hyperinflammation observed in HLH [25].

The patient’s clinical decline after COVID‐19, worsened by EBV and CMV coinfections, underscores the influence of viral triggers in uncovering genetic predispositions in FHL [10, 11, 26, 27]. SARS‐CoV‐2 hyperinflammatory effects, which resemble cytokine release syndrome, likely worsened the underlying perforin dysfunction, resulting in severe HLH manifestations [12].

The family history of early deaths (Figure 1A) aligns with patterns in FHL pedigrees, where consanguinity increases homozygous variant prevalence [12]. In our case, cosegregation analysis, where the homozygous mutation was inherited from both asymptomatic parents, aligns with autosomal recessive inheritance patterns observed in other familial HLH cases, further supporting the pathogenicity of the identified variant.

Despite intensive immunosuppressive therapy (IVIG, glucocorticoids, cyclosporine A, and dexamethasone), the patient’s fatal outcome underscores the importance of early genetic diagnosis and hematopoietic stem cell transplantation (HSCT), which is the only curative treatment for FHL2 [28, 29]. Early HSCT greatly enhances survival, especially when done before severe organ damage [30].

The ACMG classification of likely pathogenic (Table 5) is supported by rarity (PM2), conservation (Figures 2 and 3; PM1), cosegregation (PP1), in silico predictions (PP3), and HLH‐specific phenotype (PP4). However, the absence of functional assays (PS3 criterion) limits definitive causality, as in silico and structural analyses alone cannot confirm perforin’s functional impairment [13]. Future studies should incorporate NK cell cytotoxicity or perforin expression assays to validate the variant’s impact, potentially guiding the timing of HSCT [31]. This case emphasizes the diagnostic challenges of atypical HLH in older children and the critical role of genetic screening in identifying novel *PRF1* mutations. The interplay between genetic predisposition and viral triggers, particularly SARS‐CoV‐2, underscores the need for integrated genomic and immunological approaches to enhance outcomes in FHL.

**Table 5:** Summary of *PRF1* (NM_001083116.3):c.343G > A variant identified in the family.

Gene	ClinVar status	Inheritance	Zygosity (sibling)	Zygosity (parents)	Zygosity (proband)	Variant (protein)	Variant (cDNA)	ACMG classification
*PRF1*	Uncertain significant (SCV002175656.3, SCV005152109.1)	Autosomal recessive	Heterozygous	Heterozygous	Homozygous	p.Glu115Lys	NM_001083116.3:c.343G > A	Likely pathogenic

## 5. Limitations

The absence of functional assays to evaluate the impact of the p. (Glu115Lys) variants on perforin activity precludes application of the ACMG PS3 criterion. In silico predictions (Table 3), structural analysis (Figure 2B), and cosegregation data (Figure 1B) support pathogenicity, but further assays, such as NK cell cytotoxicity or protein expression assays, are required to establish causality.

## 6. Conclusion

Severe SARS‐CoV‐2 infection unmasked late‐onset FHL2 in an 11‐year‐old girl carrying the novel homozygous variant *PRF1* (NM_001083116.3):c.343G > A (p.Glu115Lys), here classified as likely pathogenic despite current ClinVar VUS status. This case underscores that primary HLH must remain in the differential diagnosis of older children with virus‐triggered hyperinflammation, particularly in consanguineous families. Prompt genetic testing for *PRF1* and related genes is essential, as HSCT offers the only curative option.

## Conflicts of Interest

The authors declare no conflicts of interest.

## Funding

This work was supported by a grant from the Isfahan University of Medical Sciences (Grant IR.MUI.MED.REC.1401.006).

## Data Availability

The data that support the findings of this study are available upon request from the corresponding author. The data are not publicly available due to privacy or ethical restrictions.
